# In Situ Preparation of Composite Scaffolds Based on Polyurethane and Hydroxyapatite Particles for Bone Tissue Engineering

**DOI:** 10.1021/acsomega.4c07673

**Published:** 2025-02-03

**Authors:** Thátila Wanessa Vieira de Sousa, Fernando da Silva Reis, Wanderson Gabriel Gomes de Melo, Aditya M. Rai, Mahendra Rai, Anderson O. Lobo, Napoleão Martins Argôlo Neto, José Milton E. de Matos

**Affiliations:** † Federal University of Piaui-UFPI, Teresina 64049-550, Brazil; ‡ Integrated Nucleus of Morphology and Stem Cell Research (NUPCelt), Postgraduate Program in Technologies Applied to Animals of Regional Interest, Federal University of Piauí, Teresina-Pi 64049-550, Brazil; § School of Management Studies, G H Raisoni University, Anjangaon Bari Rd, Badnera, Amravati, Nimbhora, Amravati 444701, India; ∥ Department of Biotechnology, Sant Gadge Baba Amravati University, Amravati 444602, India; ⊥ Laboratory of Nanostructured Oxides and Polymeric Materials - NanOPol, Chemistry Department − Nature Science Center (CCN), Federal University of Piauí, Teresina-Pi 64049-550, Brazil

## Abstract

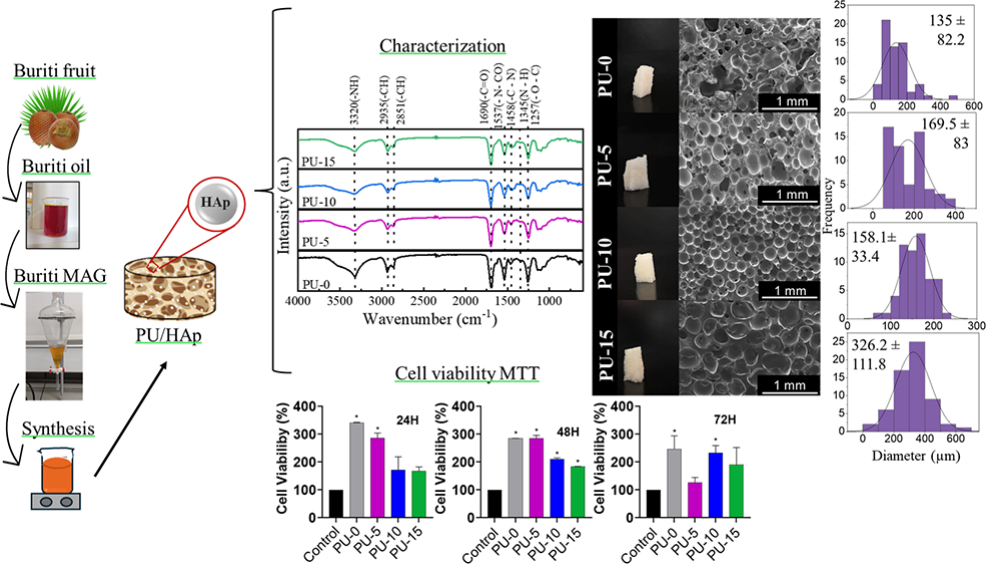

This article details the in situ preparation of composite scaffolds using polyurethane (PU) and HAp (hydroxyapatite), focusing on the unique properties of buriti oil (*Mauritia flexuosa* L.) applicable to tissue engineering. PU derived from vegetable oils, particularly buriti oil, has shown promise in bone tissue repair due to its rich bioactive compounds. Buriti oil is an excellent candidate for manufacturing these materials as it is an oil rich in bioactive compounds such as carotenoids, tocopherols, and fatty acids, which have antioxidant and anti-inflammatory properties. Furthermore, buriti oil has oleic acid as its principal fatty acid, which has been investigated as an excellent HAp dispersant. This research aimed to synthesize PU scaffolds from a polyol derived from buriti oil and incorporate HAp in different concentrations into the polymeric matrix through in situ polymerization. The chemical composition of the materials obtained, the distribution of hydroxyapatite particles in the polyurethane matrix, and the thermal stability were evaluated using Fourier transform infrared spectroscopy (FTIR), X-ray diffraction (XRD), scanning electron microscopy (SEM), energy dispersive X-ray analysis (EDS), and thermogravimetry (TGA). In addition, to investigate biocompatibility, MTT tests (3-(4,5-dimethyl-2-thiazolyl)-2,5-diphenyl-2H-tetrazolium) were conducted using rat bone-marrow-derived mesenchymal stem cells (BMMSC). Characterizations confirm the formation of PU and the presence of HAp in the polymeric matrix, and the materials did not show cytotoxicity.

## Introduction

The search for the development of new materials that contribute to the repair of bone tissue destroyed by trauma, metabolic, and degenerative diseases has stimulated the scientific–technical improvement of biomaterials to be used as bone substitutes.^1^ Treatments for critical bone damage generally require surgical interventions. The current “gold standard” treatment for this damage involves the use of an autologous bone graft, in which the graft is made with a fraction of the bone collected from the patient’s own body (usually from the pelvis or iliac crest).^2^ However, the availability of autologous bone grafts is limited and can cause serious complications, such as donor site morbidity, pain, paresthesia, prolonged hospitalization and rehabilitation, increased risk of deep infection, hematoma, and inflammation, among others.^3^

Studies have been developed using synthetic polymers in the medical field to overcome these limitations, with PU being one of the most widely used.^4^ The urethane bond forms the structure of PU, −NHCOO–, resulting from the reaction between hydroxyl groups (−OH) and isocyanate (−N=C=O), and can be synthesized in different forms, such as foams, films, and hydrogels.^5^ PU can adjust its characteristics depending on the synthesis route and precursor materials, such as polyol and isocyanate, making them versatile to meet different tissue engineering needs.^6^ This adaptability allows them to optimize their biocompatibility and promote tissue regeneration, making them an excellent option for bone repair and replacement procedures.^7^ A strategy to improve the properties of these materials, such as bioactivity and biocompatibility, is the incorporation of osteoinductive bioceramics into the polymer matrix.^8,9^ Among the studies, the use of HAp and calcium phosphates stands out, which have properties that can genetically stimulate osteoprogenitor cells and, thus, promote bone regeneration.^10^

HAp is a bioactive ceramic chemically similar to the inorganic component of bone. Its composition promotes the adhesion of bone-forming cells.^11^ Bone growth is supported by the dissolution of HAp at biological pH, producing calcium (Ca^2+)^ and phosphate (PO_4_^–3^) ions that favor cells involved in bone remodeling, mainly osteoblasts and osteoclasts.^12^ Polymer/HAp biocomposites have been widely studied in bone structures for repair and replacement of bone tissues, and PU-HAp composites have been studied/manufactured using various techniques.^10^ HAp, as a mineral compound similar to the structure of human bone, has bioactive properties that favor interaction with adjacent bone tissue, which contributes to the formation of a solid interface between the biomaterial and the bone, promoting regeneration and integration of the damaged bone tissue.^13^ Some studies report that incorporating HAp in PU biocomposites can promote significant improvements in the formation of bone tissue by osteoblasts, acting as a natural inorganic bone phase.^14^ Considering this, polymer/HAp biocomposites have great potential for clinical applications in bone tissue engineering.

Several methods are described in the literature to incorporate bioceramics into polymeric matrices.^15,16^ Some of these methods involve two steps, such as spray coating, where bioceramics can be sprayed or deposited on the surface of the polymer matrix, providing uniform coverage.^17^ In situ polymerization is a single-step method in which particles are dispersed in monomers, resulting in the simultaneous formation of a polymer composite during polymerization. The advantages of this method include better compatibility between the particle and the polymer matrix and greater dispersion of the nanoparticles.^18^

Currently, the use of vegetable oils in the preparation of polyols has aroused the scientific community’s interest, as they appear as an alternative and environmentally friendly source for the synthesis of thermosetting and thermoplastic polymers.^19,20^ Vegetable oils such as soybean, canola, rapeseed, palm, sunflower, corn, babassu, pequi, and linseed oils, as well as buriti oil, can be used.^21^ Buriti oil is a natural product derived from the fruits of the *Mauritia flexuosa* palm tree, native to the Amazon region and predominantly found in the North region. It is present in the Brazilian states of Maranhão, Piauí, Bahia, Ceará, Distrito Federal, Minas Gerais, and Mato Grosso.^22^ This oil is rich in bioactive compounds, such as carotenoids, tocopherols, and fatty acids, which have antioxidant and anti-inflammatory properties.^23^ Furthermore, it is rich in oleic fatty acid and a good HAp dispersant.^24^ Recent studies have investigated the potential of buriti oil for developing healing formulations due to its ability to accelerate the tissue regeneration process and reduce inflammation.^25,26^ These properties make buriti oil an interesting candidate for developing biomaterials with applications in tissue engineering.

Thus, the present work reports the synthesis and characterization of PU biocomposites with hydroxyapatite particles using the in situ polymerization method from a polyol derived from buriti oil aimed at its application in bone tissue engineering. The study evaluated the cytotoxicity of the materials using the MTT assay against brine shrimp (*Artemia salina*). In addition, the absorption and in vitro degradation of the materials in phosphate-buffered saline (PBS) were investigated.

## Materials and Methods

### Synthesis of Monoacylglyceride

Monoacylglyceride (MAG) was obtained as described in previous studies,^27,28^ through a reaction between buriti oil and glycerol (Contemporary Chemical Dynamics, Brazil) in a molar ratio of 1:3, in the presence of lithium hydroxide (LiOH—Vetec Fine Chemicals, Brazil) as a catalyst in a proportion of 0.05% of the oil mass. Buriti oil was purchased from the southern region of PI in the city of Sebastião. Initially, a system was prepared with a round-bottom flask in a heating bath. After stabilizing the temperature up to 70.0 ± 5.0 °C, the oil and glycerol were added, and then, the lithium hydroxide (LiOH) was added. The reaction medium was maintained at 70.0 ± 5.0 °C, and after 4 h of reaction, a separation funnel was used to separate the two phases formed and collect the MAG in the lower phase.^27^

### Synthesis of Composite Scaffolds

The PU and the bicomponent (PU/HAp) were synthesized in situ in a single step, with foam formation occurring under constant opposition at a temperature of 70.0 ± 5.0 °C. For the synthesis of PU/HAp, first, 0.75 g of MAG, 0.90 of polyethylene glycol 400 (PEG 400), and a certain amount of HAp were stirred uniformly in a beaker. Materials with 5, 10, and 15% HAp were synthesized as described in Table 1. Subsequently, hexamethylene diisocyanate (HDI) in the MAG:HDI molar ratio of 1:2.5 was added, and the system was kept under stirring until polymerization occurred.^27^ After solid formation, the sample was left for another 24 h at the same temperature to cure the polymers. The the same methodology for the PU description was followed, however, without the HAp.^28^ HAp was synthesized by the company Graphen Technology and provided by the Bioinspired Materials Laboratory—UFPI.

**Table 1:** Sample Codes, Composition, and Percentage of Hap in Composites

Sample	MAG (g)	PEG 400 (g)	HAp (g)
PU-0	0.75	0.90	0
PU-5	0.75	0.90	0.0375
PU-10	0.75	0.90	0.075
PU-15	0.75	0.90	0.112

### Characterization of Materials

Gas chromatography with a flame ionization detector (GC-FID) was used to analyze buriti oil. These oils were subjected to the transesterification procedure to improve the volatility of the fatty acids and allow their identification and quantification. A standard of methyl esters of 37 fatty acids (FAMEs) was used to quantify the fatty acids. The transesterification was carried out for oils obtained by cold pressing. For this, 200 μL of internal standard C19:0 (1000 mg L^–1^) dissolved in hexane was added to 5 mg of oil, followed by the addition of 50 μL of 2 mol L^–1^ KOH solubilized in methanol–methanolic potassium hydroxide (KOH–MeOH), and then vortexed for 30 s. At the end, the solution formed two phases, and the supernatant was transferred to Eppendorf microtubes containing an anhydrous Na_2_SO_4_ crystal, which serves as a drying agent. The microtube containing the sample was centrifuged at 10 000 rpm for 10 min at room temperature. After centrifugation, approximately 175 μL of the upper phase was transferred to amber vials capped with a Teflon septum, which were stored at −15 °C in the freezer until analysis. Subsequently, each sample and standards were analyzed by GC-FID in triplicate.

After extraction, the materials were analyzed by gas chromatography—GC equipped with a flame ionization detector—FID (GCMS-QP2010 Ultra, Shimadzu, Kyoto, Japan), and a stationary phase capillary column 50% cyanopropylphenyl-methylpolysiloxane (DB-225 ms, 15 m × 250 μm i.d. × 0.25 μm film thickness) from Agilent J&W, Santa Clara, CA, USA. The column temperature was programmed as follows: the initial temperature was maintained at 80 °C for 3 min, then increased by 5 °C min^–1^ to 180 °C, held for 3.0 min, finally increased by 3 °C min^–1^ up to 220 °C, and maintained for 5.67 min. One microliter of each sample and the FAME standard was injected in split mode (1:10), with an injector and detector temperature of 240 °C, and helium gas (99.999% purity) was used as the carrier gas at a flow rate of 1.0 mL min^–1^. The tests were carried out in 4 repetitions. FAMEs were identified by comparing retention times with those of a previously injected standard fatty acid mixture (code CRM47885; Supelco, Bellefont, PA, USA). The contribution of each compound to the mixture was calculated by the relative area (%) of its respective peak in the chromatogram. Fatty acids have been reported as their common names.

The fatty acids were confirmed by analysis using gas chromatography coupled to mass spectrometry (GC-MS), and the mass spectrometer was operated using electron ionization at 70 eV; full scan mode of analysis in the *m*/*z* range of 35 at 600, with an acquisition rate of 0.30 scans s^–1^; a delay time of 1.25 min; and a temperature of the ion source and interface of 240 °C. The methyl esters were identified by comparing the FAMES retention times and the mass fragments of the compounds registered in the National Institute of Standards and Technology (NIST) library with 80% similarity between the spectra.

The synthesized polymers were characterized by FTIR Equipment, Vertex 70 from Bruker, with 120 scans in a range of 400 to 4000 cm^–1^ and a resolution of 4 cm^–1^ in ATR mode equipped with a germanium crystal. Furthermore, they were characterized by XRD with a LABX XDR 600, Shimadzu, Cu–Kα (λ = 1.5406 Å) equipment—with 2θ in the range of 10 to 60°, a scan rate of 2° min^–1^, and an exposure time of 40 min. The diffractograms obtained were compared with profiles reported in the literature and their respective cards from the International Center for Diffraction Data database (ICDD).

The micrographs and elemental analysis were obtained using a scanning electron microscope (SEM-EDS) with a field emission gun (FEI)—model Quanta FEG 250, with an acceleration voltage from 1 to 30 kV, equipped with EDS SDD (Silicon drift detectors), Bruker model Quantax EDS, and detector XFlash 5010. The EDS analysis provided information on the distribution of the elements of interest, Ca and P, in the analyzed area. Thermal gravimetric analysis (TGA-DTG) was realized using the SDT Q600 V20.9 Build 118 20 (TA Instruments) equipment. Samples were properly weighed in alumina pans (mass of 5 ± 0.5 mg) and heated in a temperature range of 25–600 °C at 10 °C min^–1^ 119 under 10 °C min^–1^ 120 argon flux.

### The Apparent Density (Mass/Volume)

The apparent density (ρ) of the foam samples was determined by the ratio between the mass (*w*) and volume (*V*), according to the ABNT NBR 8537 standard, which establishes the method for calculating the apparent density of flexible polyurethane foams. The test was performed in triplicate, and the standard deviation is given.

### PBS Absorption and Degradation In Vitro

The PBS absorption behavior and in vitro degradation of PU were determined gravimetrically. To investigate the maximum absorption capacity of PBS, 0.1 g of each dry sample was immersed in 10 mL of PBS at room temperature. After 30 min, 2 h, 4 h, and 7 days, the swollen samples were removed from the medium, and the excess water was removed using a paper towel. Then, the PBS absorption capacity (%) for each sample was determined according to eq 1.^29,30^
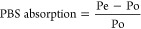
1

Po and Pe correspond to the weights of PU before and after immersion in the PBS solution, respectively.

For the in vitro degradation study, the PU samples were cut approximately in dimensions 10 mm × 5 mm × 2 mm, immersed in 10 mL of phosphate-buffered solution (PBS) (pH 7.2), and incubated at a temperature of 37 °C for 28 days. Every 7 days, samples were removed from the solution, and any water drips were removed using absorbent paper before measuring their mass. The PU was then dried at 60 ± 5 °C and the dry mass was measured. The mass percentage was determined according to eq 2.^30^
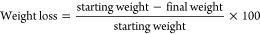
2

### Toxicity Assay

Aqueous extracts of the materials were prepared at a concentration of 10 mg mL^–1^, and after 24 h of engineering, the extracts were diluted in different concentrations of 0.1, 0.5, 1.0, and 5.0 mg mL^–1^. Initially, *Artemia salina* cysts were added to synthetic seawater, and after 48 h, the nauplii hatched. A quantity of 10 live nauplii was collected and placed in contact with aqueous extracts at concentrations of 0.1, 0.5, 1.0, and 5.0 mg mL^–1^. After 24 and 48 h, the survival rate of the nauplii was determined and the saline solution was used as a negative control.^31^

### In Vitro Cytotoxicity

To evaluate if materials have any cytotoxic effects on the rat bone-marrow derived mesenchymal stem cells (BMMSC), the MTT assay was adapted from the protocol described by Capella et al. (2019) based on the mitochondrial function through the reduction of 3-(4,5-dimethylthiazol-2-yl)-2,5-diphenyltetrazolium bromide (MTT) to a colored insoluble formazan salt. The BMMSC were seeded at 1.31 × 10^3^ cells per well in a 96-well plate, cultivated in 100 μL of DMEM (modified Eagle medium) and 15% FBS (fetal bovine serum), and incubated for 24 h. Then, 50 μL of the sample was placed over the culture and incubated for 24, 48, and 72 h. After that, the materials were removed, and the MTT assay was carried out. A control (BMMSC in usual culture medium) was included to validate the viability protocol. Then, an MTT solution (5 mg mL^–1^) was added to cells to achieve a final concentration of 0.5 mg in DMEM and incubated for 4 h. The medium was removed and replaced with 100 mL of DMSO (dimethyl sulfoxide) to dissolve the formazan crystals. The colorants’ absorbance was recorded at 570 nm in a microplate reader (Biotek Elx 800, Winooski, VT, USA). After 24 and 48 h, the viable cell number was expressed as OD (optical density) of formazan or neutral red dye obtained from cell growth in contact with materials and control groups.^32^

Statistical analyses were performed with GraphPad Prism 8 software for statistical computing using the one-way ANOVA. The comparisons were performed using Dunett’s posthoc test. The values are expressed as mean ± standard deviation (SD) and were considered significant when *p* ≤ 0.05. For *, *p* < 0.05.

## Results and Discussion

### Characterization of Composites

A GC-MS analysis with derivatization was performed to investigate the percentage of fatty acid completions in the triglyceride chains of the vegetable oil used as a precursor in synthesizing monoacylglycerols (MAGs). The analysis performed with the buriti oil revealed percentages of 77.7% for oleic acid, 19.1% for palmitic acid, and 1.23% for stearic acid, as described in Table 2. These results are in agreement with the previously published literature.^33,34^

**Table 2:** Percentage Composition of Individual Fatty Acids in Buriti Oil

Fatty acids	Oil buriti (%)
lauric acid (C12:0)	0.02 ± 0.008
myristic acid (C14:0)	0.053 ± 0.008
pentadecnoic acid (C15:0)	0.042 ± 0.003
palmitic acid (C16:0)	19.1 ± 0.5
palmitoleic acid (C16:1)	0.22 ± 0.02
magaric acid (C17:0)	0.078 ± 0.008
heptadecenoic acid (C17:1)	0.061 ± 0.008
stearic acid (C18:0)	1.23 ± 0.09
oleic acid (C18:1n-9)	77.3 ± 0.5
linoleic acid (C18:2n-6)	0.83 ± 0.02
gamma-linolenic acid (C18:3n-6)	0.57 ± 0.02
arachidic acid (C20:0)	0.075 ± 0.004
gondoic acid (C20:1n-9)	0.38 ± 0.01
behenic acid (C22:0)	0.013 ± 0.001
lignoceric acid (C24:0)	0.046 ± 0.009
AGs	20.17
AGMIs	78.46
AGPIs	1.37
AGPIs/AGs	0.07

Gas chromatography (GC) analysis of the precursor oil used to obtain the monoglyceride adds valuable information about the chemical composition of the raw material, contributing to a better understanding of the properties and behavior of the polymer.

**Figure 1:**
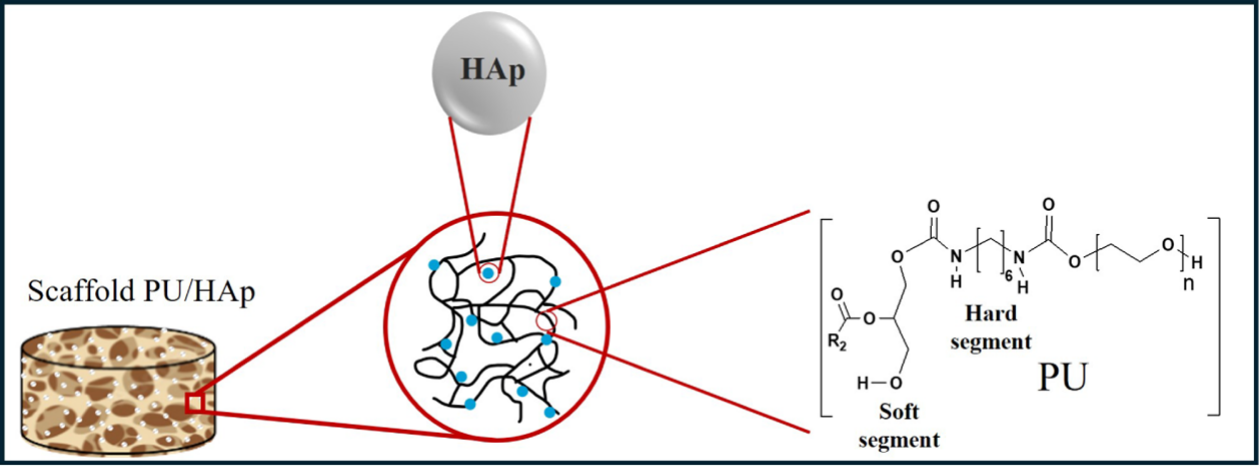
Schematic and structure of the biocomposite PU/HAp.

In this study, we aimed to fabricate PU biocomposites with hydroxyapatite particles for bone tissue engineering. The schematic and structure of PU/HAp are shown in Figure 1.

**Figure 2:**
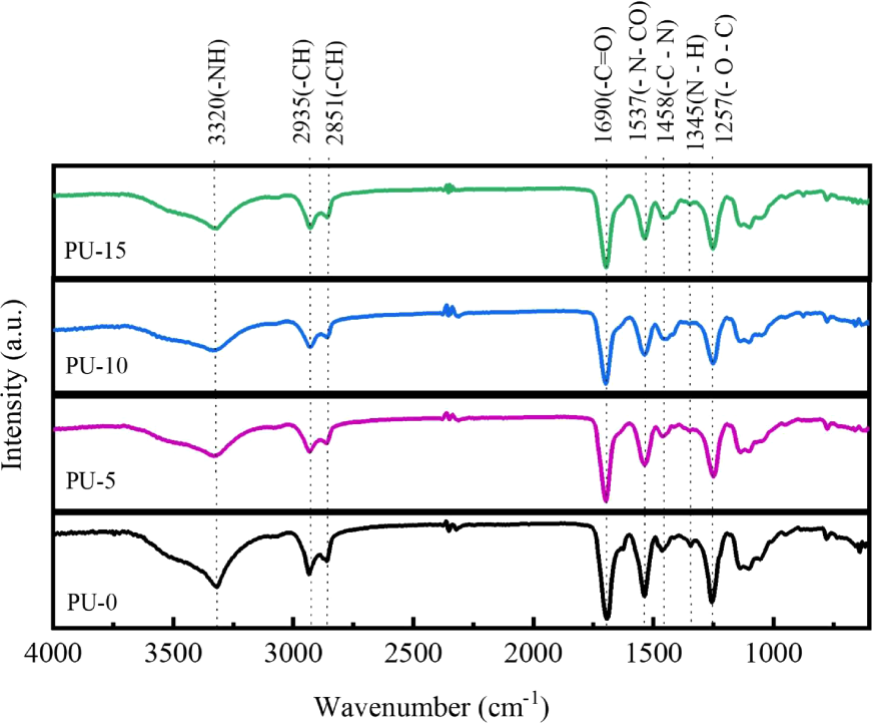
FTIR spectra of polyurethane without hydroxyapatite (PU-0) and polyurethane with 5% (PU-5), 10% (PU-10), and 15% (PU-15) of HAp.

FTIR spectra of synthesized PU are presented in Figure 2. It was possible to identify the typical characteristic bands found in the spectra of these materials, where the band at 3319 cm^–1^ corresponds to the stretching of −N–H in the urethane bond.^35^ At 2935 cm^–1^, it can be attributed to the asymmetric stretching of CH in −CH_3_ and at 2851 cm^–1^ to the symmetric stretching of CH in −CH_2_ extending to an aliphatic structure. The region at 1693 cm^–1^ corresponds to C=O stretching in urethane bonding. The corresponding band −N–CO stretching can be observed at 1537 cm^–1^ and the −C–N stretching at 1458 cm^–1^ in primary amide.^36^ The band in the region of 1345 cm^–1^ can be attributed to N–H bending, and already at 1256 cm^–1^, stretching can be attributed to −O–C stretching in urethane bonding.

Furthermore, the absorption band at 770 cm^–1^ may be related to urethane vibrations (C–O–O).^37−39^ For the graphs of materials with HAp, it is possible to observe the regions that prove the formation of the polymer. However, no significant differences were observed when compared to the PU graph, indicating that an overlap of the absorption bands may have occurred in the region of 1036 cm^–1^ corresponding to the (PO_4_)^3–^ group.^40^

**Figure 3:**
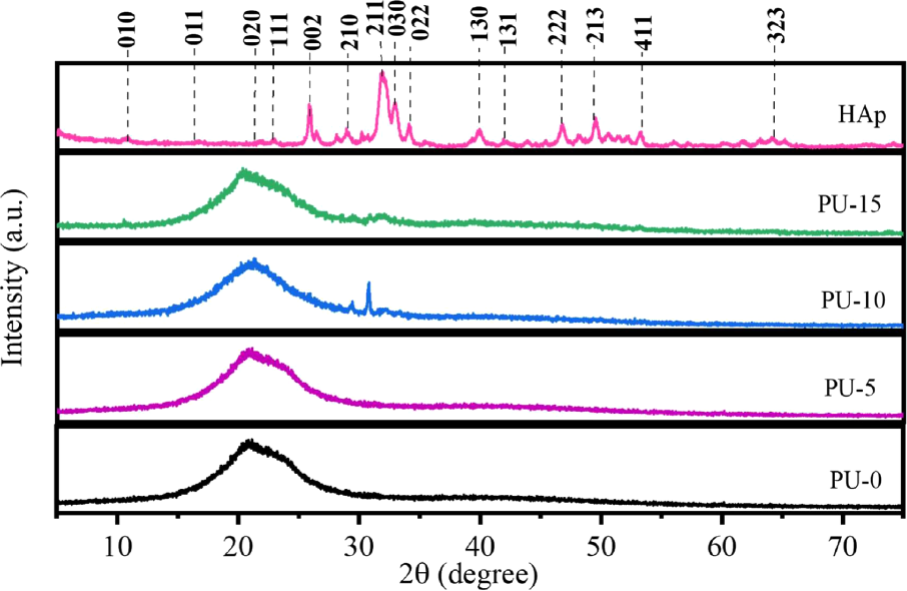
XRD patterns of polyurethane without hydroxyapatite (PU-0) and polyurethane with 5% (PU-5), 10% (PU-10), and 15% (PU-15) of HAp.

Another approach to identifying the HAp phase in the polymer matrix was to analyze the samples by X-ray diffractometry. Diffractograms of the synthesized polymers are shown in Figure 3 and reflect the amorphous nature of the polymer matrix and PU. The HAp diffractogram was compared to JCPDS 96-900-2214. Among the crystalline planes observed, the most notable were the (002), (211), (202), (310), (203), and (213) located in 2θ equivalent to 25,90°, 31,90°, 34,20°, 39,79°, 46,70°, and 49,60°. The presence of these planes indicates the existence of the HAp.^10^ In contrast, the DRX standard of PU-0 includes a broad amorphous structure, indicating little organized structure in the material.

**Figure 4:**
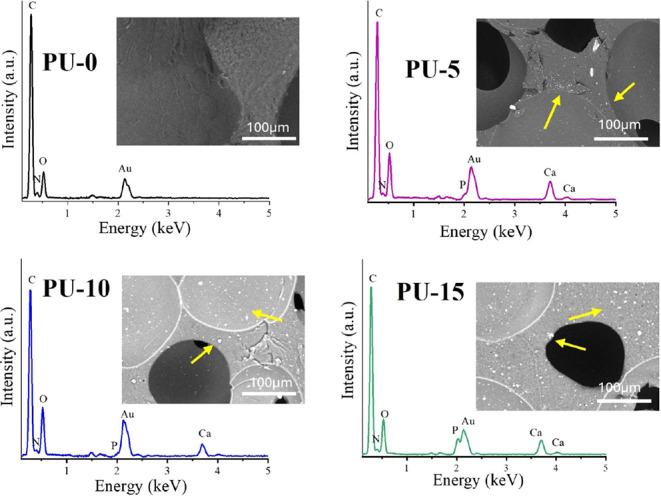
EDS of polyurethane without hydroxyapatite (PU-0) and polyurethane with 5% (PU-5), 10% (PU-10), and 15% (PU-15) of HAp.

Furthermore, the corresponding peaks of HAp in PU-10 and PU-15, identified by EDS analysis, can be observed. Figure 4 shows specific peaks for calcium (Ca) and phosphorus (P) in the EDS curves, which indicate the presence of HAp in the analyzed samples, corroborating the XRD results.

**Figure 5:**
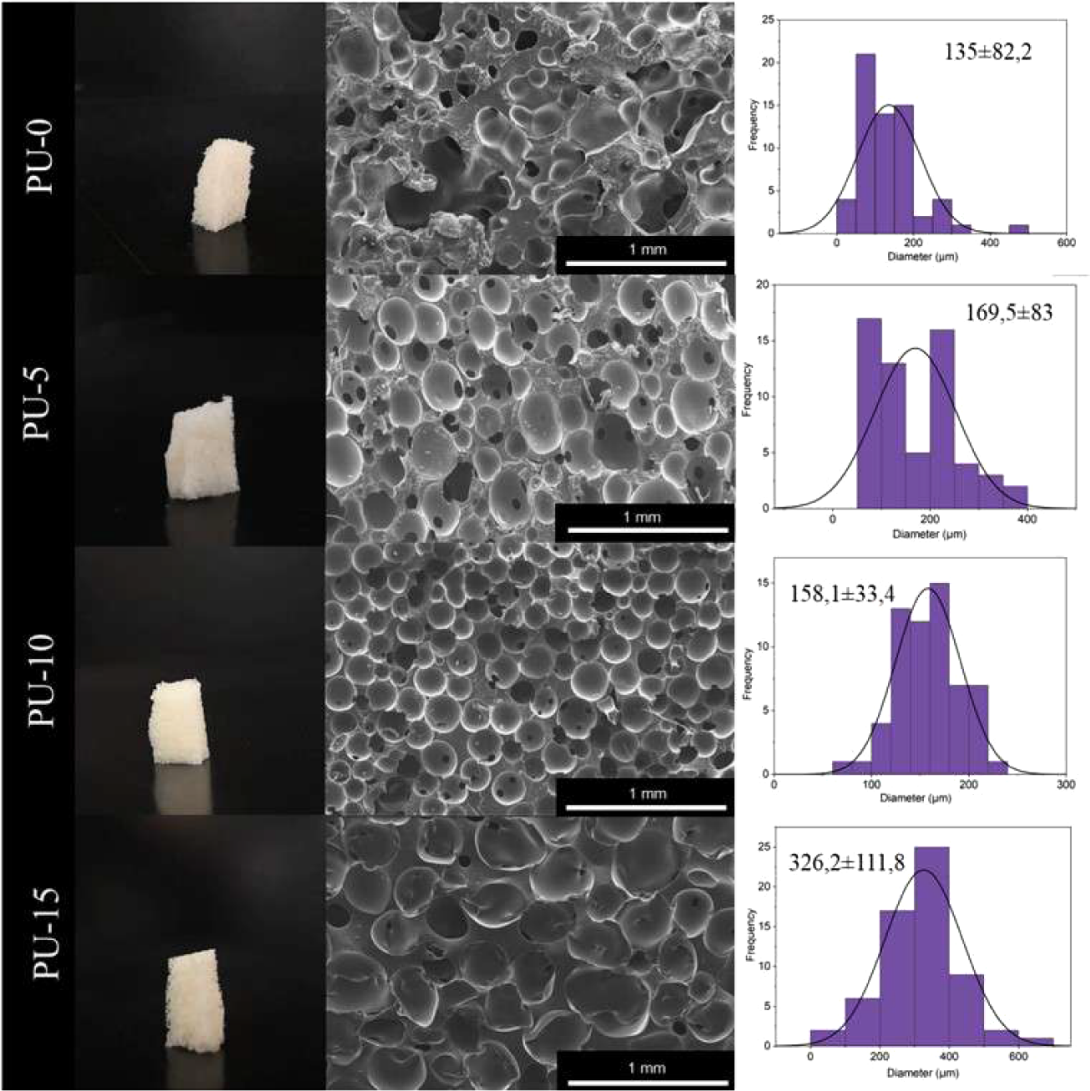
Micrography and pore size distribution of polyurethane without hydroxyapatite (PU-0), polyurethane with 5% (PU-5), 10% (PU-10), and 15% (PU-15) of HAp.

Figure 5 shows the analyzed samples’ macroscopic images (photographs), micrographs, and pore size distribution graphs. The image obtained for the PU sample shows the formation of pores with varying sizes and irregular distribution. The pure PU and biocomposite scaffolds exhibited a pore size range of 135 to 326 μm. The samples with HAp also present the formation of pores of varying sizes, whereas the sample with 10% HAp showed better uniformity in the pores, which had an average size of 158 μm, and HAp can influence the pore size.^10^ The pure PU sample had a smaller pore size; however, we can observe a high relative standard deviation, demonstrating the lack of uniformity in the pore size. By analyzing the micrographs, it is also possible to observe the presence of defects (holes) in the PU structure. The scaffolds produced with 15% HAp exhibited more open pores compared to the other scaffolds produced, with an average size of 326 μm.

The density values for materials with different percentages of hydroxyapatite (HAp) obtained are given in Table 3. It is possible to notice that the density gradually increases from PU-0 to PU-10, suggesting that the incorporation of HAp contributes to the densification of the foam. This may suggest that HAp can influence the formation of smaller and more uniformly distributed pores, contributing to a more compact and homogeneous structure.^41^

**Table 3:** Apparent Densities of the Materials Obtained

Sample	Density (g/cm^3^)	Standard deviation (*s*)
PU-0	0.370	0.037
PU-5	0.389	0.099
PU-10	0.424	0.032
PU-15	0.320	0.015

However, for PU-15, the density shows a reduction, suggesting that higher concentrations of HAp may negatively influence the density. This indicates an optimal limit for the incorporation of HAp, above which the physical properties of the foam may be compromised. The HAp particles may have formed clusters, creating heterogeneity. Furthermore, the amount of HAp in the PU-15 sample may have influenced the formation of larger pores and the formation of structural flaws.^42^

**Figure 6:**
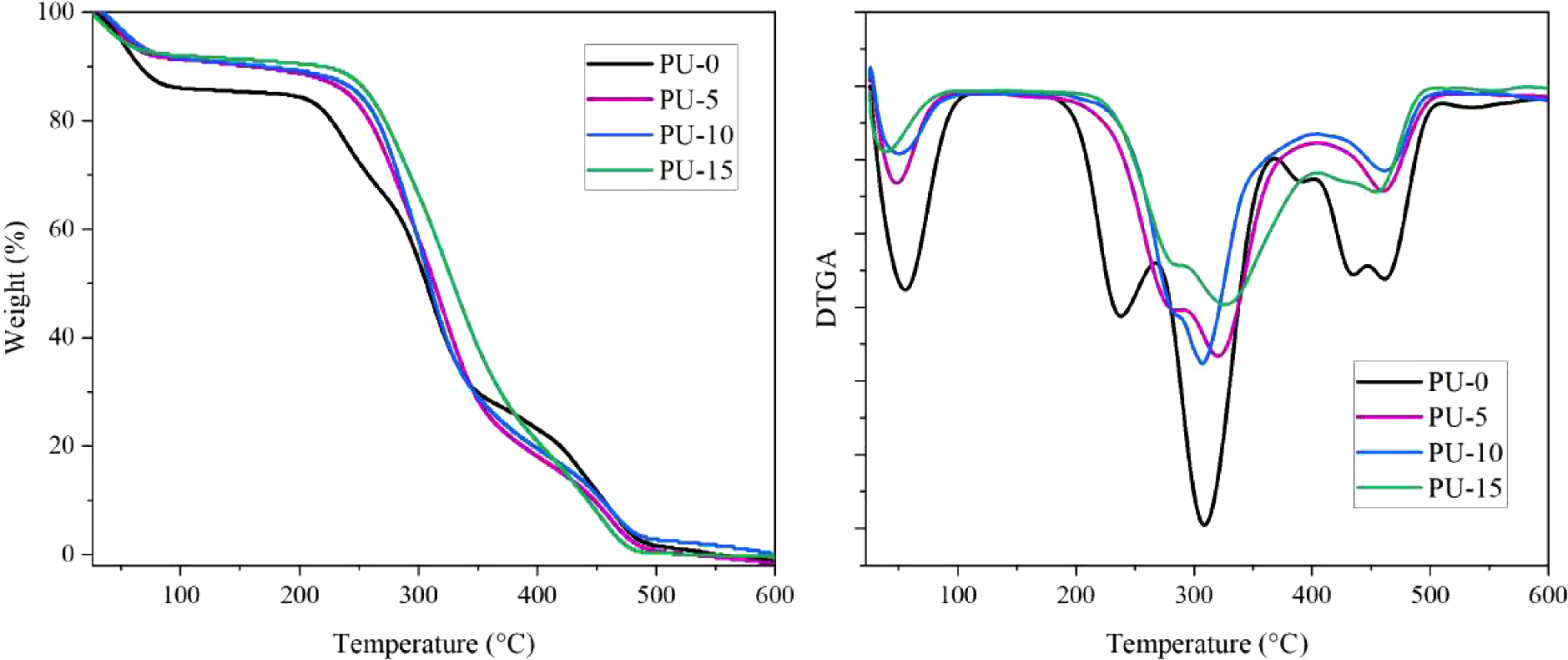
TGA and DTGA of materials of polyurethane without hydroxyapatite (PU-0) and polyurethane with 5% (PU-5), 10% (PU-10), and 15% (PU-15) of HAp.

The PU produced without and with HAp were examined from the point of view of their thermal degradation, and the TGA and DTG curves are shown in Figure 6. The PU and composite presented three main degradation processes in addition to an initial event that may be related to the evaporation of volatile components and moisture present, which was called event I. All events with their respective temperatures and percentage of mass loss are described in Table 4.

**Table 4:** Mass Loss Events Obtained in TGA Analysis of Polyurethane without Hydroxyapatite (PU-0) and Polyurethane with 5% (PU-5), 10% (PU-10), and 15% (PU-15) of HAp

Samples	Events	Temperature range (°C)	DTG peak (°C)	Weight loss (%)	Residual (%)
PU-0	I	80	56	15	-
	II	207–270	236	18	-
	III	270–346	307	37	-
	IV	346–480	462	23	7
PU-5	I	66	47.5	9	-
	II	248–287	281	17	
	III	287–348	320	40	-
	IV	348–500	460.5	31	3
PU-10	I	75	51	9	-
	II	250–290	283	23	
	III	290–339	308	39	-
	IV	339–477	462	24.3	4.7
PU-15	I	63	43	9	-
	II	253–300	281	23	
	III	300–375	329	41.6	-
	IV	375–485	460	26.4	-

For PU-0, the mass loss of about 18% that occurs within the temperature range of 207–270 °C indicates the rupture of bonds in the rigid urethane segments, which can decompose into alcohol and isocyanate.^43^ The event between the 270 and 346 °C range may be related to breaking bonds in the soft segment of urethane with the formation of primary and secondary amines and carbon dioxide.^44^ While one-step polyurethane synthesis is less controlled than multistep methods, it is still possible to produce segmented polyurethanes when using PEG and HDI in appropriate proportions, as demonstrated in previous studies.^45,46^ The last stage, in the range between 346 and 480 °C, may be related to the breaking of remaining bonds and the degradation of residues.^47^

The graph reveals that the decomposition patterns of all of the samples show similarities. Despite this, the inclusion of HAp had a positive impact on the thermal degradation resistance of the materials compared to PU-0. This finding is significant, as it suggests that the addition of HAp can enhance the thermal stability of composites, which could have wide-ranging implications in various industries. For event II, as described in Table 4, the materials PU-5, PU-10, and PU-15 presented an initial degradation temperature of 248, 250, and 253 °C, respectively, while PU-0 presented a temperature of 207 °C. The addition of HAp to PU can increase the thermal stability of the material due to the physical and chemical properties of HAp, since HAp is known for its thermal stability at high temperatures.^44,48^

### PBS Absorption and Degradation In Vitro

**Figure 7:**
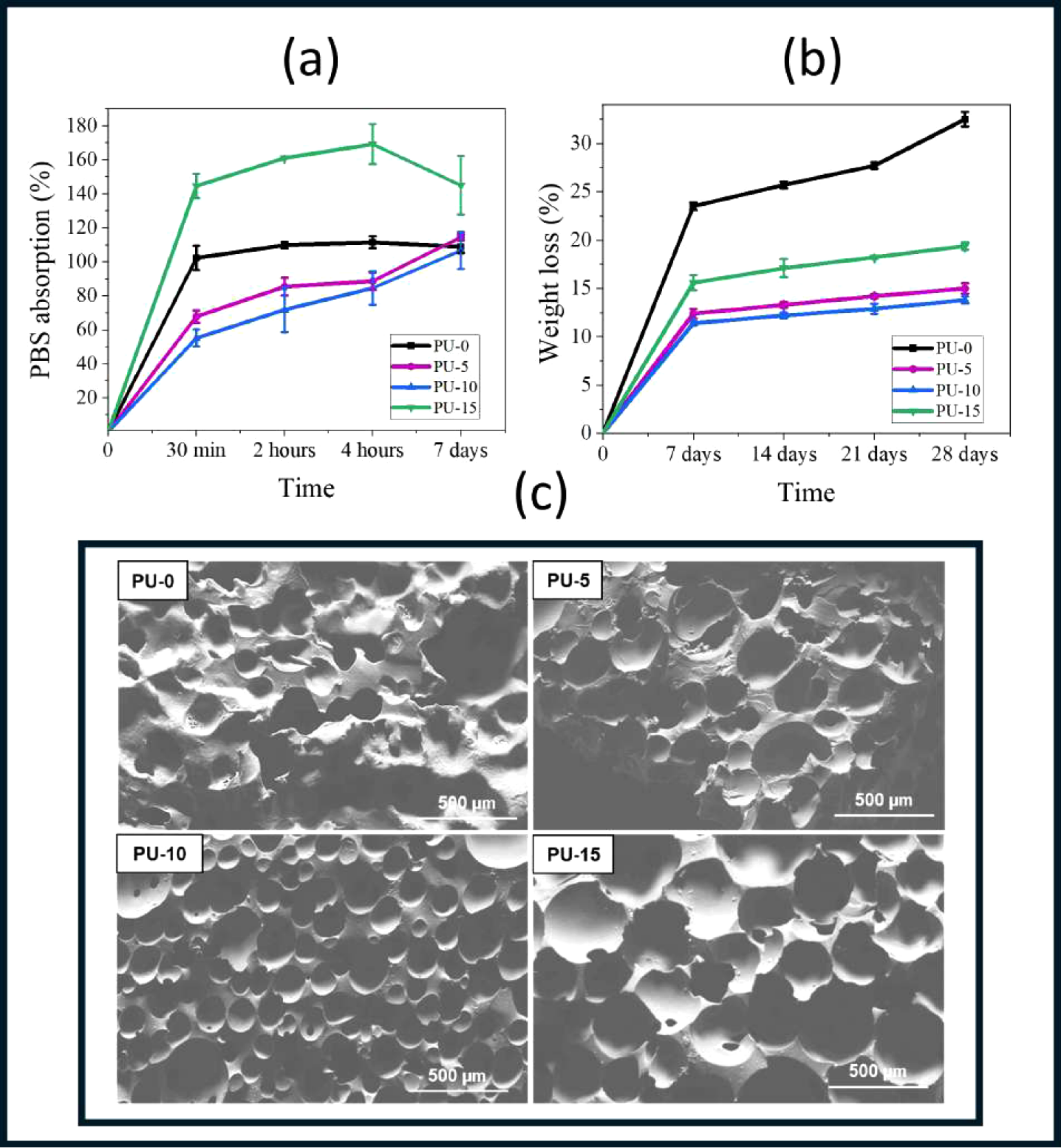
PBS absorption of the polymers synthesized at different time intervals (a). In vitro degradation of synthesized polymers (b). Micrographs of the samples studied after the degradation test (c). Polyurethane without hydroxyapatite (PU-0) and polyurethane with 5% (PU-5), 10% (PU-10), and 15% (PU-15) of HAp.

Figure 7 shows PBS absorption and in vitro degradation tests performed at different time intervals. Phosphate-buffered saline closely resembles the ionic concentration and pH of human bodily fluids, and performing these tests is important to evaluate the polymer’s performance under conditions that mimic the body’s internal environment.^49^ Analyzing the results in Figure 7a, all PUs showed high absorption of the PBS solution. In 30 min of incubation, the samples PU-10 and PU-15 showed lower and higher absorption rates, which were 55 and 135%, respectively. After 7 days of incubation, the material with 10% HAp showed lower absorption (106.5%), while the material with 15% HAp showed a higher percentage of solution absorption, which was 144.8%. In terms of absorption rate, the materials follow the following order: PU-10 < PU-5 < PU-0 < PU-15. This trend may be related to the pore size of the materials as well as their degree of porosity, since the larger the pores, the greater the amount of PBS solution penetrating the material.^50^

**Figure 8:**
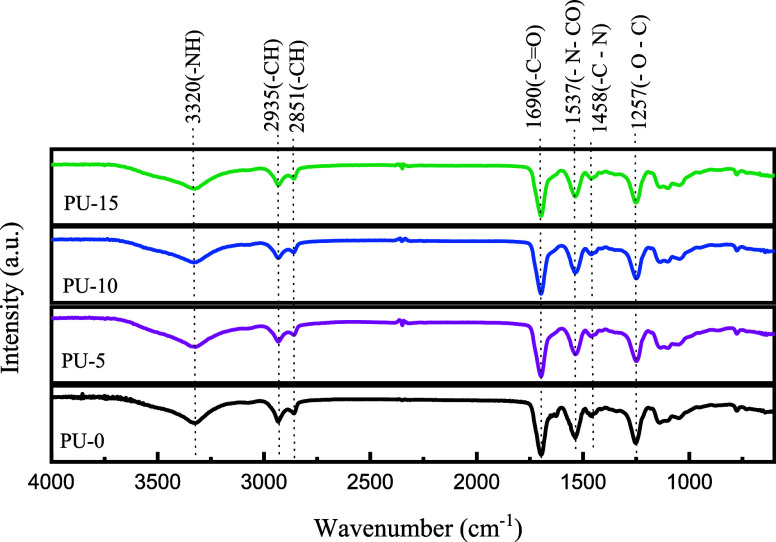
FTIR of the samples studied after the degradation test: polyurethane without hydroxyapatite (PU-0) and polyurethane with 5% (PU-5), 10% (PU-10), and 15% (PU-15) of HAp.

The polymer degradation process (Figure 7b) involves three stages: the first stage of the biodegradation process involves the absorption of water/body fluids; the second stage of degradation is known as the induction stage, during which the ester bonds in the polymer are hydrolyzed; and the third stage of degradation is the erosion stage, which causes the polymer to lose mass.^30^ As shown in Figure 8, the FTIR spectra obtained after the degradation in the test in PBS reveal a decrease in the intensity of the bands in the regions 1690 cm^–1^ (C=O) corresponding to the ester group and 1257 (O–C) and 1345 cm^–1^ (N–H) attributed to the urethane bond, breaking the biocomposite structure during the hydrolysis process.^30,51^ The degradation rate was the most significant for all materials studied in the first 7 days.

Analyzing the percentages of mass loss throughout the 28-day experiment, PU without HAp showed the highest mass loss of 32.7%. Meanwhile, PU-5, PU-10, and PU-15 recorded 14.9, 13.8, and 19.4%, respectively. Some studies suggest that water absorption is directly related to the degradation rate and that the greater the fluid absorption, the greater the degradation rate. Composites follow this trend, which does not occur with PU, indicating that HAp may have influenced the reduction in the degradation rate.^52^ Furthermore, the surface of the samples was analyzed by SEM (Figure 7c), which showed small changes after immersion in PBS solution. The samples demonstrated areas of erosion on their surface.

The ideal degradation rate for biomaterials used in bone repair may vary depending on several factors, including the type of injury, the age of the patient, the specific characteristics of the biomaterial, and the environment in which it will be implanted.^53^ However, the degradation test helps to evaluate the degradation behavior of biomaterials in PBS, serving as a tool to compare and optimize the degradation characteristics of materials.^54^

### Toxicity Assay

The *Artemia salina* assay is a widely used method to evaluate the toxicity of various substances, including biomaterials.^55^ This method is economical, simple, and rapid; requires minimal resources; and can provide valuable preliminary data on a substance’s potential toxicity.^56^ The toxicity estimate is based on the proportion of dead larvae in relation to live larvae compared with a control without toxic substances.

**Figure 9:**
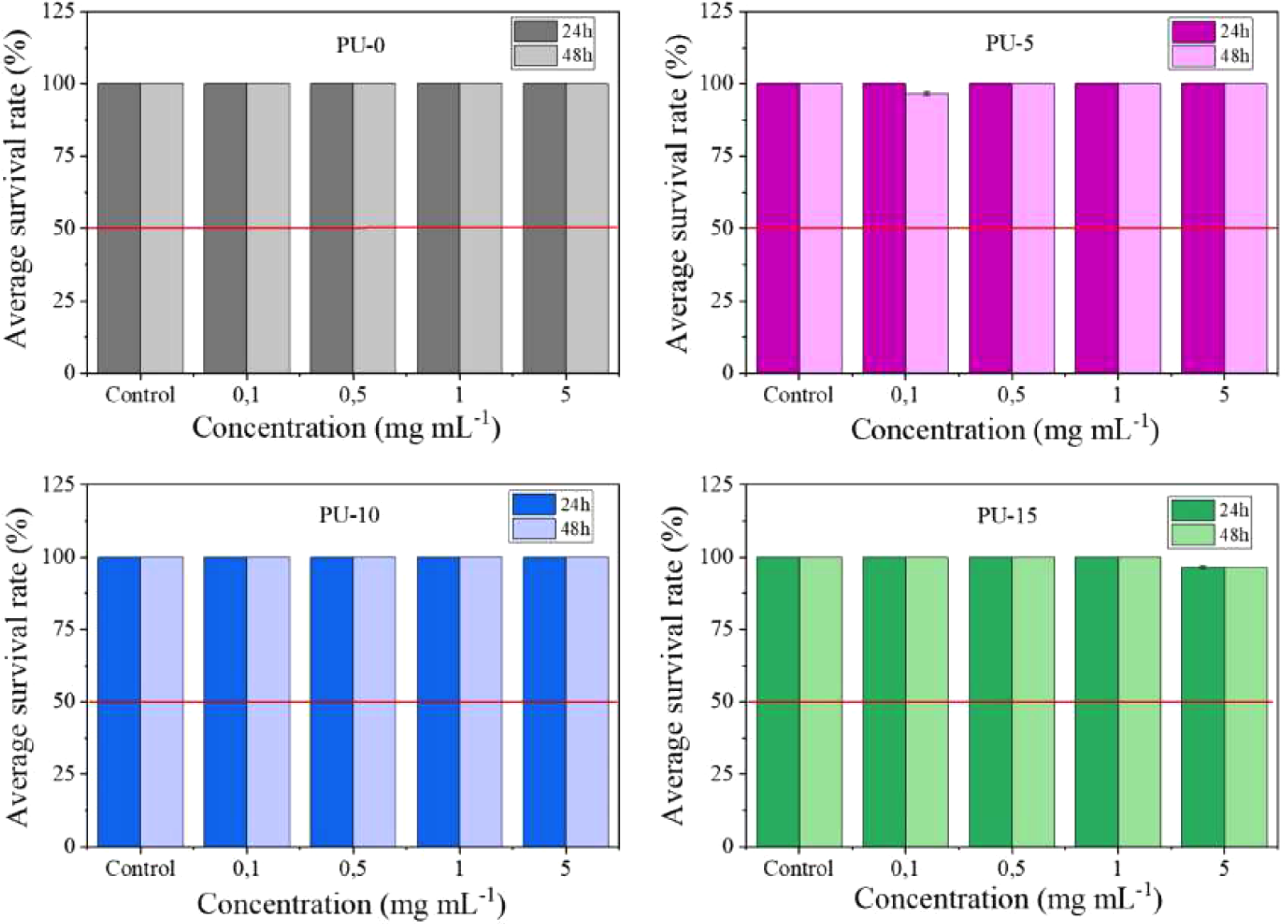
Toxicity of synthesized materials against *Artemia salina* for 24 and 48 h. Polyurethane without hydroxyapatite (PU-0) and polyurethane with 5% (PU-5), 10% (PU-10), and 15% (PU-15) of HAp.

Figure 9 shows that the materials tested, PU without HAp and PU with 5, 10, and 15% HAp, did not show toxicity at any concentration, with results higher than LD50 showing that the materials did not present toxicity at the concentrations tested. All groups had a survival rate of above 95%, which may indicate the safety of the materials tested. The ability of the obtained PU not to cause harm and to maintain a high nauplius survival rate highlights their suitability for use in a variety of medical applications.

### In Vitro Cytotoxicity

**Figure 10:**
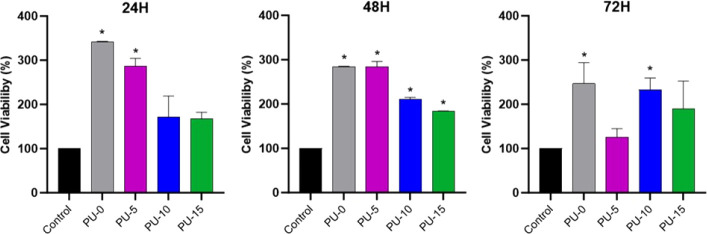
Cell viability according to the MTT assay, 24, 48, and 72 h, for the synthesized materials. Polyurethane without hydroxyapatite (PU-0) and polyurethane with 5% (PU-5), 10% (PU-10), and 15% (PU-15) of HAp.

In this study, an MTT assay was conducted to evaluate the cell proliferation rate at three time points: 24, 48, and 72 h within the structure of the synthesized scaffolds. According to the ISO standard (ISO 10993-5), the sample has a toxic effect if the viability of cells treated is less than 70% of the negative control (white), with cell death greater than 30%. Therefore, to be considered noncytotoxic, cell viability must be equal to or greater than 70%.^57^Figure 10 presents the results of the MTT assay, which indicate that all materials achieved a higher analysis rate than the control at all time points. Statistically, all materials maintained cell viability levels greater than or equal to those of the control. This suggests that the materials did not exhibit cytotoxicity, and both the PU-0 scaffold and the HAp scaffolds demonstrated a favorable structure for cell adhesion, dissemination, and proliferation without causing cytotoxic effects.^58^

The greater capacity of scaffolds to promote cell viability, compared to the control (cells + cell culture medium), can be explained by the biomaterials’ architecture.^59^ Porous structures have a larger surface area than the control, which provides more available sites for cell proliferation since they can adhere and proliferate both on the external surface and on the internal walls of the pores.^60^ This three-dimensional feature not only increases the area available for cell growth but also creates a microenvironment more similar to that found in the natural extracellular matrix, which can further stimulate biological processes.^61^

Despite the HAp scaffolds showing slightly lower cell viability values compared to those of PU-0, they still maintained acceptable levels of viability, implying that the presence of HAp does not significantly compromise cell health. At 24 and 48 h, PU-0 and PU-5 biocomposites showed significantly higher cell viability than the control, while PU-15 was statistically equal to the control. This variation in cell viability may be related to the size, pore distribution, and morphology of the HAp-modified biocomposites.^62^ Furthermore, structures with pore interconnectivity present better diffusion of nutrients, oxygen, and growth factors, facilitating cellular survival and metabolism.^60^ These results affirm the potential of both the PU-0 and HAp scaffolds for use in biomedical applications, aligning with our research goal.

## Conclusion

Using renewable resources to produce polymeric materials is an increasingly relevant approach in the search for sustainable and ecofriendly alternatives. The incorporation of HAp in different proportions in the polymeric matrix of PU produced from buriti oil offers a combination of the favorable properties of this polymer and the additional benefits provided by the presence of the mineral compound. FTIR spectroscopy confirmed the acquisition of the proposed materials in this work. Analysis of the XRD and SEM/EDS techniques revealed the presence of HAp in the polymer matrix and confirmed the successful synthesis of the polymer with HAp. The micrographs obtained by SEM revealed the porous characteristics of the analyzed materials, which is relevant for its application in bone tissue engineering, as it can contribute to cell growth in biomedical applications. The results of the PBS absorption test showed a relationship between the size of the pores and the degree of absorption of the solution; according to the data obtained with the SEM, the material with the largest average pore size was PU-15, and consequently, it was the material that presented a higher absorption rate. The degradation test showed that the polymers with HAp showed less mass loss than those without HAp. Furthermore, assessing the material’s toxicity demonstrated promising results, with a low possibility of adverse effects on living organisms. The lack of toxicity of buriti-oil-based PU, both in pure form and with the addition of HAp, reinforces their safety for use in biomedical applications.
