# Correction to “In Situ Preparation of Composite Scaffolds Based on Polyurethane and Hydroxyapatite Particles for Bone Tissue Engineering”

**DOI:** 10.1021/acsomega.5c05310

**Published:** 2025-08-22

**Authors:** Thátila Wanessa Vieira de Sousa, Fernando da Silva Reis, Wanderson Gabriel Gomez de Melo, Aditya M. Rai, Mahendra Rai, Anderson O. Lobo, Napoleão Martins Argôlo Neto, José Milton E. de Matos

**Corrections:**

We would like to correct Figure 3 from our original publication. Unfortunately, the image XRD PU-5 was incorrectly selected. Images XRD PU-5 and XRD PU-0 are slightly similar. To correct this, we have completely replaced Figure 3 with the correct data set. These corrections do not affect the integrity or conclusions of the study. And we are issuing a correction to the Author Contributions section of the original manuscript, including this information (DOI: [10.1021/acsomega.4c07673]). We apologize for the oversight.

**Figure 3:**
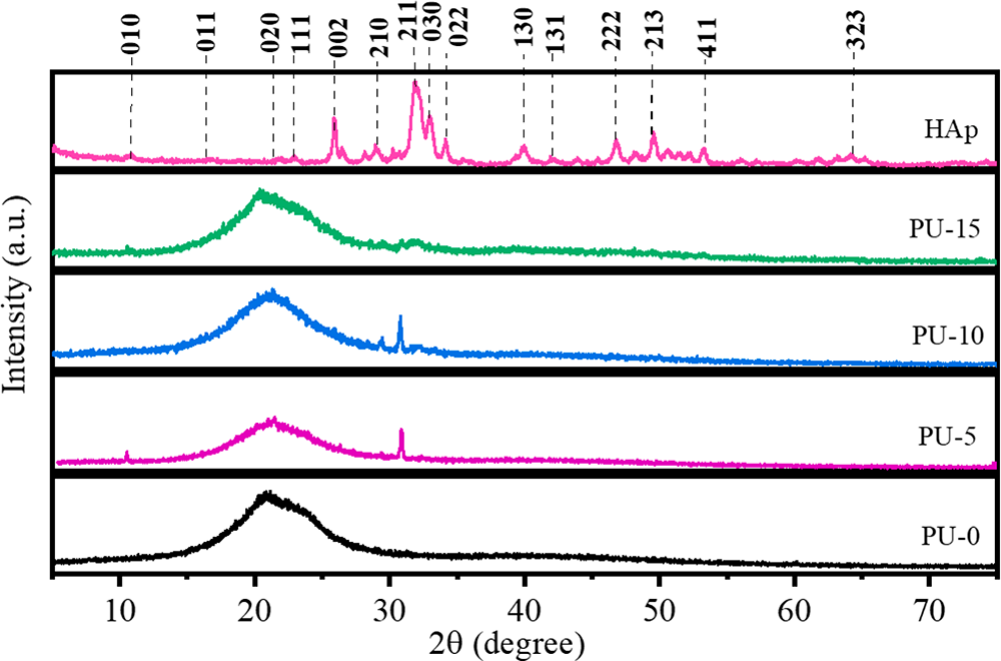
XRD patterns of polyurethane without hydroxyapatite (PU-0) and polyurethane with 5% (PU-5), 10% (PU-10), and 15% (PU-15) of HAp.

**Corrected Figure:**

